# Supplementation with *Lycium barbarum* byproducts improves energy and nitrogen metabolism, and reduces methane emissions in sheep grazing alfalfa/tall fescue pastures

**DOI:** 10.1016/j.aninu.2025.08.003

**Published:** 2025-10-06

**Authors:** Xiaoyun Zhang, Wuchen Du, Kaili Xie, Lijuan Ran, Wanhe Zhu, Fujiang Hou

**Affiliations:** State Key Laboratory of Herbage Improvement and Grassland Agro-Ecosystems, Ministry of Agriculture and Rural Affairs, College of Pastoral Agriculture Science and Technology, Lanzhou University, Lanzhou 730020, Gansu, China

**Keywords:** *Lycium barbarum* byproduct, Grazing sheep, Nitrogen utilization, Energy utilization, Methane emission

## Abstract

Functional native herbage (FNH), rich in bioactive components and secondary metabolites, holds potential for enhancing the productivity of grazing livestock and mitigating methane (CH_4_) emissions. The processing of *Lycium barbarum* yields byproducts rich in flavonoids, polysaccharides, betaine, and other bioactive compounds, which may serve as effective livestock feed additives. This study aimed to assess the effects of *L. barbarum* byproducts on digestion, metabolism, and CH_4_ emissions in sheep grazing on sown pastures in northwest China. Twenty-four 6-month-old male sheep with similar body conditions were selected and randomly assigned to four groups (*n* = 6) based on dietary treatments: an unsupplemented control group (CON), a 2.5% *L. barbarum* seed (LBS)-supplemented group, a 7.5% *L. barbarum* residue (LBR)-supplemented group, and a 2.5% *L. barbarum* twigs (LBT)-supplemented group. The experiment lasted for a total of 75 d. Digestive metabolism experiments and respiratory gas measurement tests were conducted. Compared to the CON group, supplementation with 2.5% *L. barbarum* seed or 7.5% *L. barbarum* residue increased (*P*< 0.001) dry matter intake, ether extract intake, gross energy intake, and improved (*P*< 0.001) energy utilization efficiency, including digestible energy, metabolizable energy, and net energy. Compared to the CON group, the LBS group, live weight gain increased by 44.44% (*P* = 0.018), accompanied by 27.37% and 30.56% reductions in net energy requirements for maintenance and metabolizable energy requirements for maintenance, respectively. Additionally, daily CH_4_ emissions and CH_4_ emissions per unit grass area were reduced (*P* < 0.05) by 20.34% and 20.43%, respectively. In the LBR group, live weight gain increased by 33.33% (*P* = 0.018), accompanied by 46.03% and 30.56% reductions in net energy requirements for maintenance and metabolizable energy requirements for maintenance, respectively. Daily CH_4_ emissions and CH_4_ emissions per unit grass area were reduced (*P* < 0.05) by 22.75% and 22.78%, respectively. Compared to the CON group, the live weight gain in the LBT group remained largely unchanged (*P* > 0.05), while daily CH_4_ emissions and CH_4_ emissions per unit of grass area were reduced by 17.05% (*P* < 0.05) each. The judicious application of *L. barbarum* byproducts in livestock production can enhance the productivity of grazing sheep, optimize resource utilization, reduce CH_4_ emissions, ameliorate the ecological environment, and foster the sustainable development of animal husbandry.

## 1 Introduction

The global demand for livestock products continues to rise, intensifying pressure on limited land and water resources and presenting challenges related to sustainable production and environmental impact (FAO, 2018; Grumbine et al., 2021). Efficient utilization of feed resources is essential not only for maximizing livestock productivity but also for improving energy and nitrogen (N) utilization, both of which are crucial for animal growth and reducing nutrient losses and greenhouse gas emissions in ruminant production systems (Hristov et al., 2011; Place et al., 2011). Improved energy and N retention directly contribute to greater feed efficiency and sustainable intensification, while mitigating methane (CH_4_) emissions. Methane is a potent greenhouse gas with a global warming potential substantially higher than CO_2_ (Gerber et al., 2013; Johnson and Johnson, 1995), and represents a significant dietary energy loss (2% to 12% of gross energy intake) for the animal (Hristov et al., 2013). Concurrently, inefficient dietary N utilization reduces protein conversion and leads to excessive N excretion, which poses an environmental concern (Calsamiglia et al., 2010). As such, dietary interventions that optimize energy and N utilization and simultaneously reduce CH_4_ emissions are a priority for sustainable livestock systems (Beauchemin et al., 2020).

Recently, there has been a surge in interest in utilizing native plants rich in bioactive substances as potential new feed additives to improve animal production and reduce CH_4_ emissions (Somboonchai et al., 2022; Wanapat et al., 2018; Zhang et al., 2020, Zhang et al., 2020). The consumption of plants rich in flavonoids, carotenoids, and other bioactive compounds has been shown to enhance immunity in sheep (Khan et al., 2012). Substances such as plant oils, polysaccharides, alkaloids, tannins, flavonoids, and phenolic compounds have demonstrated the ability to improve rumen function and microbial community structure, leading to improved animal performance, energy and N utilization, and reduced CH_4_ emissions (Chikwanha et al., 2019; Huyen et al., 2012; Iannaccone et al., 2018; Manasri et al., 2012; Patra et al., 2017; Phesatcha et al., 2013; Tayengwa et al., 2020).

*Lycium barbarum* is widely cultivated in arid regions globally, particularly in China and other Asian countries (Amagase et al., 2011). In China, approximately 76,400 to 114,600 tons of residual fruits/pomace, 11,500 tons of seeds, and over 200,000 tons of discarded branches/leaves are generated annually (Ministry of Agriculture and Rural Affairs of the People's Republic of China, 2022; Gonçalves et al., 2017; Masci et al., 2018; Ma et al., 2022). As both a medicinal and edible plant, *L. barbarum* is abundant in nutrients and active substances (Konarska, 2018). Its active components include polysaccharides (3% to 10%), flavonoids (0.1% to 1.0%), carotenoids (0.03% to 0.50%), anthocyanins (0.02% to 0.10%), and more (Islam et al., 2017; Liu et al., 2014; Wang et al., 2010a, Wang et al., 2010b; Wang et al., 2005; Zeng et al., 2023). It was hypothesized that supplementing grazing sheep with *L. barbarum* byproducts can improve production performance and reduce CH_4_ emissions. Thus, this study aims to assess the impact of *L. barbarum* byproducts as a sheep feed supplement on two critical performance indicators: (1) livestock performance, by monitoring average daily live weight gain, feed intake, and digestibility, and (2) environmental sustainability, by monitoring CH_4_ and CO_2_ emissions, as well as N and energy metabolism.

## 2 Materials and methods

### 2.1 Animal ethics statement

The experiments were conducted following the guidelines established in the experimental field management protocols (file NO. 2010-1 and 2010-2), which were approved by the Animal Use and Care Committee of Lanzhou University (Lanzhou, Gansu, China). All animal procedures were performed in accordance with the Guidelines for the Care and Use of Laboratory Animals of Lanzhou University and were approved by the Animal Ethics Committee of Lanzhou University: SCXK (gan) 2018-0002. All procedures for handling and caring for animals conform with China's regulations on the protection and use of laboratory animals (GB/T 35892-2018, China National Standard, 2018), and are approved by the Chinese Zoological Society.

### 2.2 Test site

The study was conducted from June to August 2021 at Linze Pratacultural Research Station of Lanzhou University (100°02′E, 39°15′N), located in Linze County, Zhangye, Gansu, China. The region, characterized by its four distinct seasons and temperate continental climate, has an annual average temperature ranging from 7.7 to 9.3 °C. Precipitation is predominantly concentrated between July and September, with an annual average of 112.9 to 118.4 mm, against an evaporation rate of 1830.4 mm. The area primarily utilizes a specialized intensive cropping production system in conjunction with an extensively integrated crop-livestock production system (Hou et al., 2008; Zhang et al., 2022). *L. barbarum* byproducts (seeds, residues [fruit pulp residues after juice extraction], and twigs [discarded young stems and leaves from annual pruning]) were procured from Ningxia Saishang Hongbaozhu Agricultural Technology Co., Ltd. (Ningxia, China). The main active ingredients, including polysaccharides, total flavonoids, and betaine, along with their nutritional values, are detailed in Table 1.

**Table 1:** The nutritional level of the sown pastures forage, *Lycium barbarum* seed*,* residue, and twigs (%, as DM basis).

Item	Seed	Residue	Twigs	Sown pastures forage
**Nutritional levels**				
OM	96.82	93.69	93.73	88.42
CP	14.67	18.17	5.48	13.84
EE	16.23	7.07	3.40	3.43
NDF	45.08	17.40	73.53	55.94
ADF	22.03	3.46	47.48	33.31
ME, MJ/kg	13.94	14.01	13.39	13.54
**Functional ingredients**				
Polysaccharide	2.84	3.64	1.93	-
Total flavonoids	0.09	0.13	0.26	-
Betaine	0.02	0.02	0.33	-

DM = dry matter; OM = organic matter; CP = crude protein; NDF = neutral detergent fiber; ADF = acid detergent fiber; EE = ether extract; ME = metabolizable energy; - = not detected.

### 2.3 Experimental animals and group design

A cohort of 24 healthy male F1 hybrid sheep (Small Tail Han Sheep × Hu Sheep crossbreed), aged six months (180 ± 7 d) with uniform body weight (34.14 ± 1.20 kg) and physiological condition, were stratified into four groups (*n* = 6) using a randomized block design. Dietary treatments comprised: (1) Control (CON), basal diet + 50 g soybean meal; (2) *L. barbarum* seeds (LBS), basal diet + 2.5% (22.5 g/d) *L. barbarum* seeds + 50 g soybean meal; (3) *L. barbarum* residues (LBR), basal diet + 7.5% (67.5 g/d) *L. barbarum* residues + 50 g soybean meal; (4) *L. barbarum* twigs (LBT), Basal diet + 2.5% (22.5 g/d) *L. barbarum* twigs +50 g soybean meal. The basal diet was alfalfa/tall fescue mixed in equal proportions, sown pasture's forage, with an approximate proportion of 55% alfalfa and 45% tall fescue determined through a standardized quadrat sampling method at the start of the experiment. The detailed description of this method can be found in section 2.4. Investigation of pasture vegetation composition. The specific proportions of *L. barbarum* byproducts (seeds, residue, and twigs) were determined through preliminary in vitro experiments conducted prior to the main study (unpublished data). The amount of *L. barbarum* byproducts supplement is calculated based on the dry matter intake (DMI) of 900 g/d according to the Chinese Standard for Meat Sheep Raising (2021). *L. barbarum* byproducts were processed into dried and pulverized samples, and were homogenized with soybean meal prior to supplemental feeding to sheep. This formulation ensured complete consumption of the byproducts, thereby eliminating selective feeding behavior and residual feed accumulation (Xun et al., 2012; Zhang et al., 2022). The nutrient levels of both forage and *L. barbarum* byproducts are detailed in Table 1.

The experiment lasted for a total of 75 d and consisted of two phases: a 15-d pre-feeding period and a 60-d main trial period. During a 15-d pre-feeding period, sheep were ad libitum grazed in the alfalfa/tall fescue sown pastures from 07:00 to 19:00 daily. Post-grazing, sheep were relocated to individual enclosures and provided with their respective *L. barbarum* byproducts supplements via limit bars. Supplementary feeds were administered at 19:30, ensuring complete consumption before releasing sheep for free movement.

As described by Wang et al. (2021), the main trial comprised of two sequential phases. (1) Metabolic testing: conducted over 6 d (2-d adaptation + 4-d sampling). Sheep were housed in individual metabolic cages with visual contact to minimize stress. Basal diet (fresh forage cut daily from grazing plots) was provided thrice (07:00, 13:00, and 19:00), and *L. barbarum* byproducts supplements were administered post-evening feeding (19:30). CON group sheep were fed soybean meal only. (2) Gas production measurement: conducted over 3 d (1-d adaptation + 2-d sampling) utilizing a medium-sized multichannel animal respiration and metabolism measurement device. This device comprises six independent sealed chambers, each housing a metabolic cage. The sheep were confined within these metabolic cages, allowing them to see one another through transparent glass. Feeding management mirrored the metabolic testing. Upon completion of the gas production measurement, the sheep are returned to the flock for grazing, mirroring the conditions of the pre-feeding period.

All sheep remained healthy throughout the experiment and returned to their pre-experimental conditions to continue their normal lives post–experiment.

### 2.4 Investigation of pasture vegetation composition

The botanical composition of the basal diet, specifically the approximate proportion of alfalfa and tall fescue, was determined through vegetation surveys using the quadrat sampling method (Bonham, 2013; Ken, 2011). The procedure involved the following steps:

Quadrat establishment and sampling design. Standardized sampling units were established using square polyvinyl chloride (PVC) quadrats measuring 0.5 m × 0.5 m. Multiple quadrats were randomly placed throughout the grazing area at the beginning of the experimental period.

Data collection. Within each quadrat, the number of individual alfalfa and tall fescue plants was recorded separately.

Calculation of proportions. The average proportion of alfalfa and tall fescue was calculated by first determining the density (plants per unit area) for each species in every quadrat. For each quadrat, the relative proportion of alfalfa was computed as the number of alfalfa plants divided by the total number of plants (alfalfa + tall fescue) within that quadrat, expressed as a percentage. This was similarly done for tall fescue. The overall proportion for the entire pasture was calculated by averaging the relative proportions across all sampled quadrats. Mathematically, this can be expressed as Coulloudon et al. (1999). For a single quadrat:

Relativeproportionofalfalfa(%)=[Numberofalfalfaplants/Totalnumberofplants(alfalfa+tallfescue)]×100;

For the entire pasture:

$$\text{Mean}\;\text{proportion}\;\text{of}\;\text{alfalfa}=\left[\sum_{i=1}^{n}\left(\text{Relative}\;\text{proportion}\;\text{of}\;\text{alfalfa}\;\text{in}\;\text{quadrat}\;i\right)\right]/n\text{,}$$

where *n* is the number of quadrats sampled.

### 2.5 Sample collection and processing

At the commencement and conclusion of the experiment, each sheep was weighed in the morning after an overnight fast. The change in body weight throughout the experimental period was recorded as live weight gain (LWG).

During the metabolic testing, the weight of forage offered to each sheep daily was recorded using an electronic scale (ACS-30KG-728, Quan Fu Industry, Shanghai, China) and designated as forage allowance. Prior to the subsequent day's feeding, residual forage remaining in the corresponding feed trough was removed, collected, and weighed to determine the previous day's forage refusal. Concurrently, representative samples of both the offered and refused forage were collected daily and dried to determine dry matter (DM) content. Following conversion to a DM basis, the difference between the forage allowance and forage refusal was recorded as DMI. Fecal and urinary excretion were collected daily from each sheep using a total collection method, implemented as follows: feces and urine excreted by the sheep passed through the metabolic cage floor onto a slightly inclined, leak-proof collection tray. Urine flowed directly down the inclined tray into a plastic basin positioned at the tray's distal end. Feces were retained on the tray surface. The urine collection basin was covered with a 2 mm mesh screen to prevent fecal contamination. Each morning, prior to feeding, the urine collection basin was carefully removed and replaced with a clean basin for the subsequent day's collection. All feces, including those retained on the floor, tray, and screen, as well as any feces deposited around the perimeter of the metabolic cage, were swept up and collected. The net weight of the collected urine and feces, after subtracting the container weight, was recorded as the daily fecal and urinary excretion. Daily, 150 g of fresh fecal samples were collected per sheep, oven-dried, and analyzed for nutrient composition. Additionally, 20 g of fresh feces were homogenized with 2 mL of 10% sulfuric acid (H_2_SO_4_) for N fixation, then stored at −20 °C for subsequent analysis of fecal N (FN) content. For urinary analysis, 20 mL of fresh urine was mixed with 10% (v/v) diluted sulfuric acid to adjust the pH < 3, and the acidified samples were stored at −20 °C for determination of urinary N (UN) and urinary energy (UE).

Greenhouse gas emissions were measured over a 3-d period using the medium-sized multichannel animal respiration and metabolism measurement device (GLIRI-MORMC-002, Grassland-Livestock-Interaction Research Institute [GLIRI], Lanzhou University, Lanzhou, Gansu, China), after which the average daily gas production was calculated.

### 2.6 Sample analysis

#### 2.6.1 Feed quality analysis

Fresh grass and fecal samples were collected and dried in an oven (DHG-9240A, Jing Hong Laboratory Instrument Co., Ltd., Shanghai, China) at 65 °C until a constant weight was reached, after which the DM content was determined. The dried samples were ground and passed through a 1 mm sieve for the determination of the nutritional quality of the samples. The neutral detergent fiber (NDF) content and acid detergent fiber (ADF) content (method 973.18; AOAC, 2002) were determined using the filter bag technique and a semiautomatic fiber analyzer (A2000I 174, Ankom Instrument Co., Ltd., Macedon, NY, USA). The gross energy (GE) of the forage, manure and urine samples was analyzed by an automatic oxygen bomb calorimeter (PARR6400, Parr Instrument Co., Hillsboro, OR, USA) (Yang et al., 2018). The total N concentration (method 990.03; AOAC, 2006) was analyzed using a Kjeldahl N 168 analyzer (Model K9840, Jinan Hanon Instruments Co., Ltd., Jinan, Shandong, China). Crude protein (CP) levels (method 990.03; AOAC, 2002) were analyzed via flow injection (FIAstar 5000 Analyzer, FOSS Analytical A/S, Hilleroed, Denmark). Crude ash (Ash) (method 990.03; AOAC, 1990) were determined by a muffle furnace (SX-G03173, Tianjin Zhonghuan Electric Furnace Co., Ltd., Tianjin, China), which required charring on an electric heating plate followed by ashing in a muffle furnace at 550 °C, after which the ash was weighed and measured. Organic matter (OM) content is calculated as the percentage of the sample excluding ash content. Ether extract (EE) (method 920.39; AOAC, 1998) was determined by a fully automated fat meter (ANKOMXT15 Extractor, Ankom Instrument Co., Ltd., Macedon, NY, USA). The nutrient content of *L. barbarum* byproducts was assessed using the forage determination method. Additionally, high-performance liquid chromatography was employed to analyze the active ingredients, including polysaccharides (Agilent Eclipse XDB-C18 column [4.6 mm × 250 mm, 5 μm], Agilent Technologies Inc., Santa Clara, CA, USA) (Yang et al., 2024; Zhang et al., 2021), total flavonoids (Zorbax SB-C18 [4.6 mm × 250 mm, 5 μm], Agilent Technologies Inc., Santa Clara, CA, USA) (Li et al., 2024; Wang et al., 2004), and betaine (Partisil SCX-10 [4.5 × 250 mm, 10 μm], Thermo Fisher Scientific Inc., Hemel Hempstead, UK) (Chendrimada et al., 2002), in three *L. barbarum* byproducts.

Metabolic energy of feed was calculated as follows:

Metabolicenergy(MJ/kgDM)=13.97−0.0127×ADF(g/kgDM)+0.0165×EE(g/kgDM)−0.0057×Crudeash(g/kgDM).

The following formulae (Cui et al., 2023; Yang et al., 2020) were used:

$$\text{Nutrient}\;\text{intake}\;\left(g/d\right)=\text{Diet}\;\text{nutrient}\;\text{concentration}\;\left(g/\text{kg}\;\text{DM}\right)\times \text{DMI}\;\left(\text{kg}/d\right)\text{;}$$

$$\text{Apparent}\;\text{digestibility}\;\left(\%\right)=100\times \left[\text{Nutrient intake}\;\left(g/d\right)-\text{Fecal}\;\text{nutrient}\;\left(g/d\right)\right]/\mathrm{Nutrient}\mathrm{intake}\;\left(g/d\right)\text{;}$$

$$\text{NI}\;\left(g/\text{day}\right)=\text{CP}\;\left(g/\text{kg}\right)\times \text{DMI}\;\left(\text{kg}\right)/6.25\text{;}$$

$$\text{UN}\;\left(g/\text{day}\right)=\text{Urine}\;\text{volume}\;\left(L/d\right)\times \text{N}\;\text{concentration}\;\text{in urine }\left(g/L\right)/1000$$

$$\text{FN}\;\left(g/d\right)=\text{Daily}\;\text{fecal}\;\text{output}\;\left(\text{kg}/d,\text{wet}\;\text{weight}\right)\times \text{Nitrogen}\;\text{concentration in feces}\;\left(g/\text{kg}\;\text{DM}\right)\times \text{DM}\;\text{content}\;\text{of}\;\text{feces}\;\left(\%\right)\text{;}$$

Digestiblenitrogen(DN,g/day)=NI(g/day)–FN(g/day);

Retainednitrogen(RN,g/day)=NI(g/day)–FN(g/day)–UN(g/day);

Grossenergyintake(GEI,MJ/day)=GE(MJ/kgDM)×DMI(kg);

$$\text{UE}\;\left(\text{MJ}/d\right)=\text{Urine}\;\text{volume}\;\left(L/d\right)\times \text{Energy concentration in urine}\;\left(\text{MJ}/L\right)\text{;}$$

$$\text{FE}\;\left(\text{MJ}/d\right)=\text{Daily}\;\text{fecal}\;\text{output}\;\left(\text{kg}/d,\text{wet}\;\text{weight}\right)\times \text{Energy concentration in feces}\;\left(\text{MJ}/\text{kg}\;\text{DM}\right)\times \text{DM}\;\text{content}\;\text{of}\;\text{feces}\;\left(\%\right)\text{;}$$

Digestibleenergy(DE,MJ/day)=GEI(MJ/day)−FEoutput(MJ/day);

$$\text{Methane}\;\text{energy}\;\left({\text{CH}}_{4}\text{–}E,\text{MJ}/\text{day}\right)={\text{CH}}_{4}\;\text{emission}\;\left(L/\text{day}\right)\times 39.54\;\left(\text{kJ}/L\right)\times 10^{-3}\text{;}$$

$$\text{Metabolizable}\;\text{energy}\;\left(\text{ME},\text{MJ}/\text{day}\right)=\text{DE}\;\left(\text{MJ}/\text{day}\right)\;–\;\text{UE}\;\left(\text{MJ}/\text{day}\right)\;–\;{\text{CH}}_{4}-E\;\left(\text{MJ}/\text{day}\right)\text{;}$$

GE and CP represent the energy and protein content in the DM of feed units, respectively.

$$\text{Heat production (HP,}\;\left(\text{MJ}/\text{day}\right)=[16.18\times O_{2}\;\text{consumption}\;\left(L/\text{day}\right)+5.02\times {\text{CO}}_{2}\;\text{emission}\;\left(L/\text{day}\right)–\;2.17\times {\text{CH}}_{4}\;\text{emission}\;\left(L/\text{day}\right)–\;5.99\times \text{UN}\;\text{excreted}\;\left(g/\text{day}\right)\text{]}\times 10^{-3}\text{;}$$

$$\mathrm{Energy}\mathrm{balance}(E_{g},\;\text{MJ}/\text{kg}\;{\text{BW}}^{0.75}\text{)}=\text{ME}\;\left(\text{MJ}/\text{kg}\;{\text{BW}}^{0.75}\right)-\text{HP}\;\left(\text{MJ}/\text{kg}\;{\text{BW}}^{0.75}\right)\text{;}$$

(1) $$E_{g}=a_{1}\text{ME}\;\text{intake}\;–\;b_{1}$$

(2) $$\text{HP}=a_{2}\text{ME}\;\text{intake}\;–\;b_{2}$$

Eqs. (1), (2) were used for data fitting. The constant (*b*_*1*_ or *b*_*2*_) was used as the net energy requirement for maintenance (NE_m_). Metabolizable energy requirement for maintenance (ME_m_) was calculated from the constant divided by the slope (*b*_*1*_/*a*_*1*_) in Eq. 1.

#### 2.6.2 Determination of CH_4_ and CO_2_ concentrations

This study utilized a medium-sized, multi-channel animal respiratory metabolism measurement device (GLIRI-MORMC-002, GLIRI). The device comprised of six independent indirect open-circuit breathing chambers, each housing one sheep. CO_2_ and CH_4_ production, along with O_2_ consumption, for each sheep were reported as a 2-d average. The breathing chambers, measuring 1.98 m in length, 1.46 m in width, and 1.68 m in height, were constructed with a steel frame and were enclosed on the sides and top with transparent plexiglass. The base was securely fixed to the ground with poured cement, which was treated with a layer of waterproof material to ensure proper sealing. A breathable plastic flooring was installed over the cement floor. Each breathing chamber was equipped with a small door on one side, allowing entry and exit for both sheep and staff, as well as a feeding window on the opposite side. Both the small door and the feeding window were fitted with latex seals that ensure a complete seal when closed. Additionally, holes of uniform size were incorporated into the lower part of the side deflection and the top of the breathing chamber, through which pipes for gas exchange were installed. Ventilation facilitated by an air pump, which creates a slight negative pressure within the breathing chamber. The temperature and humidity within the chamber were maintained at 15 to 20 °C and (30 ± 10)%, respectively, within the appropriate range. Each breathing chamber equipped with a gas flow meter (GFM57, Aalborg Instruments & Controls Inc., Orangeburg, NY, USA) to measure the gas flow rate. The flow rate set between 6 and 10 Nm^3^/h, ensuring that the concentrations of CO_2_, CH_4_, and O_2_ in the air remained within the manufacturer's recommended measurement ranges for accurate assessments. The gas in the breathing chamber was sampled by a multi-channel gas sampler (YA-03DLQ ten-channel multichannel gas sampler, Yi'an Technology Co., Ltd., Shenzhen, Guangdong, China) into a gas analyzer (VA-3000, Horiba Stec Co., Kyoto, Japan). The gas analyzer alternately measured the concentration of CO_2_, CH_4_, and O_2_ in the gas entering and leaving the single port channel of each breathing chamber and the concentration of the three gases in the air at an interval of 21 min. Each breathing chamber and atmosphere was continuously measured for 3 min. The daily cleaning of the breathing chamber and the daily feeding of grass were carried out by trained personnel within a fixed time range immediately after the measurement of each breathing chamber and ensuring that the work was completed before the next round of measurement. All gases passed through three filtering devices before entering the gas analyzer ensuring that the particles entering the analyzer did not exceed 5 μm. Prior to each test, the gas analyzer was calibrated using standard gas (N_2_ without O_2_ and known amounts of CO_2_, CH_4_, and O_2_; Dalian Special Gas Co., Ltd., Dalian, Liaoning, China) to determine the concentrations of CH_4_, CO_2_, and O_2_ in the atmosphere, which were measured within the absolute ranges of 0 to 200 μL/L, 0 to 2000 μL/L, and 0 to 25% (v/v), respectively. Before and after the test, the breathing chambers were calibrated by releasing known amounts of analytical grade CH_4_, CO_2_, and O_2_ into each chamber. The gas recovery rate was measured and determined to be (100 ± 2)%. The production of CH_4_ and CO_2_, as well as the consumption of O_2_ by the sheep, was calculated by multiplying the cumulative gas flow rate in the respective breathing chamber by the difference in concentration of each gas in the samples entering and leaving the chamber. Detailed parameters of the metabolic chamber and the device used are provided in Wang et al. (2020) and Liu et al. (2020).

### 2.7 Statistical analysis

All experimental data were organized using Microsoft Excel 2021, and all subsequent analyses were conducted using SPSS 20.0. Data were evaluated for homogeneity of variances and normality using the Shapiro–Wilk test. With strict control over consistent test conditions between batches, a one-way analysis of variance (ANOVA) was employed to evaluate and exclude the effects of test batches on DMI, fecal and urinary excretion, and greenhouse gas emissions (Table S1), with the batches as a fixed effect and sheep within batches as a random effect, and each sheep serving as a test unit. The statistical model is as follows:

$$Y_{ij}=\mu +T_{i}+C_{j}+\varepsilon_{ij}\text{,}$$

where *Y*_*ij*_, animal observation; *μ*, overall mean; *T*_*i*_, fixed effect of batches (*i* = 1-4); *C*_*j*_, random effect of sheep (*j* = 1-6); and *ε*_*ij,*_ random error. Results were presented as least squares means for treatment, standard error of the mean, and *P*-values for the effect of treatment. A *P*-value of less than 0.05 was considered statistically significant, while a *P*-value of less than 0.10 was considered to indicate a statistically significant result.

The intake and digestibility data of nutritional indicators among groups were analyzed using one-way ANOVA, with differences between means evaluated using Duncan's multiple range test. With the treatment group as a fixed effect and sheep within treatment as a random effect, each sheep served as a test unit. The statistical model is as follows:

$$Y_{ij}=\mu +T_{i}+C_{j}+\varepsilon_{ij}\text{,}$$

where *Y*_*ij*_, animal observation; *μ*, overall mean; *T*_*i*_, fixed effect of treatment (*i* = 1-4); *C*_*j*_, random effect of sheep (*j* = 1-6); and *ε*_*ij*_, random error.

Results are presented as least squares means for treatment, standard error of the mean, and *P*-values for the effect of treatment. A *P*-value of less than 0.05 indicates a statistically significant difference, while a *P*-value between 0.05 and 0.10 suggests a trend.

Linear regression within the SPSS 20.0 software was applied to analyze the correlation between nutrient intake, LWG of grazing sheep, and CH_4_ emission, CH_4_-E, fecal energy (FE), UE, and FN emissions per unit metabolic body weight of sheep. The following model was used to test the normality of residuals:

$$Y=a+a_{1}X_{1}+b_{2}X_{2}+b_{3}X_{3}+\ldots +b_{n}X_{n}\text{,}$$

where *Y*, N, energy and CH_4_ emissions per unit metabolic body weight of sheep; *a*, general coefficient; *a*_*1*_ *… a*_*n*_, standardized coefficient of nutrient intake or LWG; and *X*_*1*_ *… X*_*n*_, specific nutrient intake or LWG. In the test of the inter-subject effect, if the *P*-value is less than 0.05, it is appropriate. If it is greater than 0.05, it is necessary to delete the index greater than 0.05 until equation *P*-value is less than 0.05.

## 3 Results

### 3.1 Supplementing feed with *L. barbarum* by products to grazing sheep: Effects on intake, digestion, and LWG

Supplementation with *L.* *barbarum* seeds significantly increased LWG compared to the CON group (*P* = 0.018, Table 2). Both LBS and LBR groups demonstrated substantial improvements in nutrient intake and digestibility relative to the CON group. Specifically, compared to the CON group, the LBS group showed 44.56% (*P* < 0.001), 56.15% (*P* < 0.001), 64.76% (*P* = 0.001), and 65.63% (*P* < 0.001) increases in DMI, ether extract intake (EEI), neutral detergent fiber intake (NDFI), and acid detergent fiber intake (ADFI), respectively. Similarly, compared to the CON group, the LBR group displayed elevated DMI (45.89%, *P* < 0.001), EEI (59.84%, *P* < 0.001), crude protein intake (CPI, 23.26%, *P* = 0.010), NDFI (55.68%, *P* = 0.001), and ADFI (61.12%, *P* < 0.001) (Table 2). In contrast, the LBT group exhibited marked reductions in nutrient intake and digestibility. Compared to LBS and LBR groups, LBT sheep displayed significant decreases in metabolic body weight-adjusted DMI (56.53% and 57.98%, *P* < 0.001), EEI (64.22% and 74.89%, *P* < 0.001), NDFI (75.36% and 65.19%, *P* = 0.001), and dry matter digestibility (DMD, 30.85% and 23.28%, *P* = 0.004). Additionally, LBT sheep showed 44.44% (*P* = 0.018), 31.58% (*P* = 0.049), 31.13% (*P* = 0.029), and 48.92% (*P* = 0.022) reductions in LWG, basal diet intake, neutral detergent fiber digestibility (NDFD), and acid detergent fiber digestibility (ADFD) compared to the LBS group, with CPI also being 30.71% lower than the LBR group (*P* = 0.010) (Table 2).

**Table 2:** Effects of the addition of *Lycium barbarum* seed, residue, and twigs on the live weight gain (LWG), nutrient intake and digestibility of sheep.

Item	Treatments^1^				SEM	*P-*value
	CON	LBS	LBR	LBT		
LWG, kg/d	0.09^b^	0.13^a^	0.12^ab^	0.09^b^	0.019	0.018
Basal diet intake, kg/d	0.98^ab^	1.00^a^	0.83^ab^	0.76^b^	0.038	0.049
DMI, kg/d	1.03^b^	1.52^a^	1.52^a^	0.98^b^	0.064	<0.001
**Nutrient intake, g/kg·BW^0.75^**						
DM	71.35^b^	103.14^a^	104.09^a^	65.89^b^	4.463	<0.001
OM	70.54	79.60	85.36	64.08	4.718	0.840
EE	2.44^b^	3.81^a^	3.90^a^	2.32^b^	0.178	<0.001
CP	8.77^b^	9.86^ab^	10.81^a^	8.27^b^	0.314	0.010
NDF	36.09^b^	59.46^a^	56.18^a^	34.01^b^	3.247	0.001
ADF	21.34^b^	35.34^a^	34.38^a^	17.22^b^	2.119	<0.001
**Nutrient digestibility, %**						
DM	64.80^a^	72.96^a^	68.74^a^	55.76^b^	0.019	0.004
OM	71.33	73.37	70.61	67.29	0.021	0.839
EE	59.38^ab^	81.35^a^	67.28^ab^	48.99^b^	0.045	0.044
CP	76.44	70.18	77.96	75.35	0.013	0.184
NDF	58.77^ab^	68.07^a^	64.68^ab^	51.91^b^	0.021	0.029
ADF	56.23^ab^	64.08^a^	56.52^ab^	43.03^b^	0.026	0.022

DM = dry matter; OM = organic matter; CP = crude protein; NDF = neutral detergent fiber; ADF = acid detergent fiber; EE = ether extract; DMI = dry matter intake; SEM = standard error of the mean.

Within the same row with different lowercase letters are significantly different (*P* < 0.05).

1 CON, the control group (no supplementation); LBS, 2.5% *L. barbarum* seeds group (22.5 g); LBR, 7.5% *L. barbarum* residue group (67.5 g); LBT, 2.5% *L. barbarum* twigs group (22.5 g); *n* = 6.

### 3.2 Supplementing feed with *L. barbarum* by products to grazing sheep: Effects on N metabolism

**Fig. 1:**
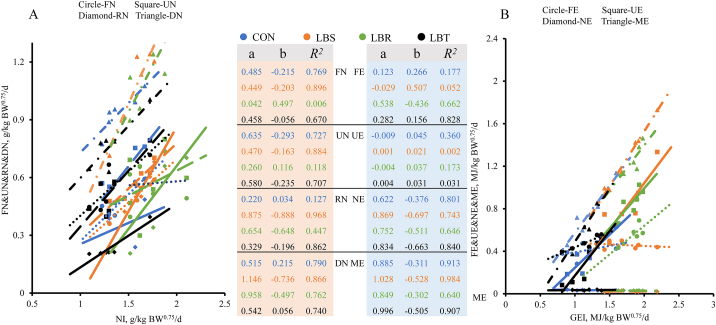
Effects of *Lycium barbarum* byproducts addition on energy and nitrogen utilization in grazing sheep. (A) Nitrogen metabolism and intake. (B) Energy metabolism and intake. In the linear regression model *y* = *ax* + *b*, the parameter a (slope) indicates the expected change in the dependent variable associated with a one-unit increase in the independent variable. The parameter b (intercept) represents the estimated value of the dependent variable when the independent variable is zero. Colors represent groupings: blue, CON (no supplementation); orange, LBS (2.5% *Lycium barbarum* seeds group); green, LBR (7.5% *L. barbarum* residue group); black, LBT (2.5% *L. barbarum* twigs group). Different colors and the same type of lines correspond to the same indicator under different treatments. FN = fecal nitrogen; UN = urinary nitrogen; RN = retained nitrogen; DN = digestible nitrogen; FE = fecal energy; UE = urinary energy; NE = net energy; ME = metabolizable energy.

Dietary supplementation with *L. barbarum* byproducts significantly influenced N metabolism in grazing sheep per unit metabolic body weight (Table 3). Sheep in the LBR group exhibited a 23.57% and 31.06% (*P* = 0.010) increase in nitrogen intake (NI) compared to the CON and LBT groups, respectively. In contrast, the LBT group demonstrated a reduction of more than 50% (*P* = 0.016) in RN and decreases of 38.96% and 50.65% (*P* < 0.05) in DN relative to the LBS and LBR groups (Table 3). As NI in sheep increased, the FN, UN, RN, and DN per unit of metabolic body weight in each group followed a similar pattern. FN and UN increased more rapidly in the CON group (FN_CON_ = 0.4852NI - 0.215, *R*^2^ = 0.769; UN_CON_ = 0.6345NI - 0.2931, *R*^2^ = 0.727), while DN and RN were higher in the LBS group (DN_LBS_ = 1.1456NI - 0.7355, *R*^2^ = 0.866; RN_LBS_ = 0.8753NI - 0.8882, *R*^2^ = 0.968) (Fig. 1A).

**Table 3:** Effects of the addition of *Lycium barbarum* seed, residue, and twigs on nitrogen metabolism in sheep.

Item	Treatments^1^				SEM	*P-*value
	CON	LBS	LBR	LBT		
**Nitrogen balance, g/kg·BW^0.75^/d**						
NI	1.40^b^	1.58^ab^	1.73^a^	1.32^b^	0.050	0.010
FN	0.47	0.51	0.57	0.55	0.022	0.463
UN	0.63	0.58	0.57	0.52	0.030	0.752
RN	0.31^ab^	0.49^a^	0.51^a^	0.24^b^	0.039	0.016
DN	0.93^ab^	1.07^a^	1.16^a^	0.77^b^	0.047	0.010
**Nitrogen utilization efficiency, %**						
FN/NI	33.02	32.59	33.25	41.47	1.460	0.081
UN/NI	44.31	36.66	32.78	40.00	1.670	0.080
RN/NI	22.67	30.76	33.97	18.54	2.373	0.068

NI = nitrogen intake; FN = fecal nitrogen; UN = urinary nitrogen; DN = digestible nitrogen; RN = retained nitrogen; SEM = standard error of the mean.

Within the same row with different lowercase letters are significantly different (*P* < 0.05).

1 CON, the control group (no supplementation); LBS, 2.5% *L. barbarum* seeds group (22.5 g); LBR, 7.5% *L. barbarum* residue group (67.5 g); LBT, 2.5% *L. barbarum* twigs group (22.5 g); *n* = 6.

### 3.3 Supplementinb feed with *L. barbarum* by products to grazing sheep: Effects on energy metabolism

Supplementing the diet with *L. barbarum* byproducts significantly impacts energy metabolism in grazing sheep. The LBS group showed significant increases in GEI, DE, ME, net energy (NE), and the ratio of ME to DE by 47.90%, 67.95%, 70.67%, 128.05%, and 3.26%, respectively, compared to the CON group (*P* < 0.001); and significant decreases in UE, UE to GE ratio, and CH_4_-E to GE ratio by 100%, 142.4%, and 84.62%, respectively (*P* < 0.05, Table 4). Additionally, the LBR group exhibited significant increases in GEI, FE, DE, ME, NE, and the ratio of ME to DE by 53.78%, 36.59%, 64.10%, 66.67%, 135.89%, and 2.60%, respectively, and significant decreases in CH_4_-E, UE to GE ratio, and CH_4_-E to GE ratio by 23.48%, 45.54%, and 93.45%, respectively, compared to the CON group (*P* < 0.05, Table 4). Furthermore, the LBT group had a significant reduction of 19.39% in CH_4_-E levels compared to the CON group (*P* < 0.05, Table 4). With the intake of total energy, the NE (NE_LBS_ = 0.8687GEI - 0.6966, *R*^2^ = 0.743) and retained energy (RE_LBS_ = 1.028GEI - 0.5278, *R*^2^ = 0.983) per unit of metabolic body weight in the LBS group increased more rapidly than in other groups. Conversely, the FE (FE_LBS_ = −0.0286GEI + 0.5037, *R*^2^ = 0.052) per unit of metabolic body weight in the LBS group decreased more rapidly than in other groups. The UE per unit of metabolic body weight in the LBR group (UE_LBR_ = −0.0036GEI + 0.0366, *R*^2^ = 0.173) decreased more rapidly than in other groups (Fig. 1B). Moreover, the net energy requirements for maintenance (NE_m_) for sheep in the CON, LBS, LBR, and LBT groups were 0.349, 0.274, 0.239, and 0.347 MJ/kg BW^0.75^, respectively, while the metabolizable energy requirements for maintenance (ME_m_) were 0.423, 0.324, 0.305, and 0.454 MJ/kg BW^0.75^, respectively (Table 5).

**Table 4:** Effects of the addition of *Lycium barbarum* seed*,* residue, and twigs on energy metabolism in sheep.

Items	Treatments^1^				SEM	*P-*value
	CON	LBS	LBR	LBT		
**Energy balance, MJ/kg·BW^0.75^/d**						
GEI	1.19^b^	1.76^a^	1.83^a^	1.14^b^	0.079	<0.001
FE	0.41^b^	0.46^ab^	0.55^a^	0.48^ab^	0.017	0.024
UE	0.04^a^	0.02^b^	0.03^a^	0.04^a^	0.001	<0.001
CH_4_-E	0.06^a^	0.05^b^	0.05^b^	0.05^b^	0.001	0.005
DE	0.78^b^	1.31^a^	1.28^a^	0.66^b^	0.074	<0.001
ME	0.75^b^	1.28^a^	1.25^a^	0.63^b^	0.075	<0.001
HP	0.38	0.45	0.39	0.43	0.022	0.663
NE	0.37^b^	0.83^a^	0.86^a^	0.19^b^	0.076	<0.001
**Energy utilization efficiency, %**						
FE/GE	35.36^ab^	26.53^b^	29.99^b^	43.11^a^	1.867	0.003
UE/GE	3.03^a^	1.25^b^	1.65^b^	3.15^a^	0.205	<0.001
DE/GE	64.64^ab^	73.47^a^	70.01^a^	56.89^b^	1.867	0.003
ME/GE	61.61^ab^	72.22^a^	68.37^a^	53.74^b^	2.036	0.002
NE/GE	29.62^ab^	46.59^a^	47.08^a^	14.25^b^	3.934	0.002
HP/GE	32.00^ab^	25.63^ab^	21.29^b^	39.49^a^	2.232	0.013
CH_4_-E/GE	0.05^a^	0.03^b^	0.03^c^	0.04^ab^	0.003	<0.001
ME/DE	95.17^b^	98.27^a^	97.64^a^	94.17^b^	0.448	<0.001

GEI = gross energy intake; FE = fecal energy; UE = urinary energy; CH_4_-E = methane energy; DE = digestible energy; ME = metabolizable energy; HP = heat production; NE = net energy; SEM = standard error of the mean.

Within the same row with different lowercase letters are significantly different (*P* < 0.05).

1 CON, the control group (no supplementation); LBS, 2.5% *L. barbarum* seeds group (22.5 g); LBR, 7.5% *L. barbarum* residue group (67.5 g); LBT, 2.5% *L. barbarum* twigs group (22.5 g); *n* = 6.

**Table 5:** The linear regression Eq. and the derived net energy (NE_m_) and metabolizable energy (ME_m_) requirements for the maintenance of sheep.

Treatments^1^	Eq.	*R*^2^	NE_m_	ME_m_	Eq. NO.
CON	E_g_ = 0.816ME intake - 0.349	0.979	0.349	0.423	(1a)
	HP = 0.184ME intake + 0.349	0.700	0.349		(1b)
LBS	E_g_ = 0.845ME intake - 0.274	0.998	0.274	0.324	(2a)
	HP = 0.155ME intake + 0.274	0.944	0.274		(2b)
LBR	E_g_ = 0.782ME intake - 0.239	0.964	0.239	0.305	(3a)
	HP = 0.218ME intake + 0.239	0.835	0.239		(3b)
LBT	E_g_ = 0.765ME intake - 0.347	0.969	0.347	0.454	(4a)
	HP = 0.235ME intake + 0.347	0.746	0.347		(4b)

E_g_ = energy balance; ME = metabolizable energy; HP = heat production.

1 CON, the control group (no supplementation); LBS, 2.5% *L. barbarum* seeds group (22.5 g); LBR, 7.5% *L. barbarum* residue group (67.5 g); LBT, 2.5% *L. barbarum* twigs group (22.5 g); *n* = 6.

### 3.4 Supplementing feed with *L. barbarum* by products to grazing sheep: Effects on greenhouse gas emissions

Supplementing the diet with *L. barbarum* byproducts significantly influenced greenhouse gas emissions in grazing sheep. In the LBS group, CH_4_ emissions, CH_4_ per DMI (CH_4_/DMI), CH_4_ per metabolic body weight (CH_4_/BW^0.75^), CH_4_ per DMD (CH_4_/DMD), and CH_4_ emissions per unit of grassland area decreased significantly by 20.41%, 80.71%, 23.22%, 39.27%, and 20.43%, respectively (*P* < 0.05, Table 6). Furthermore, in the LBR group, CH_4_ emissions, CH_4_/DMI, CH_4_/BW^0.75^, CH_4_/DMD, and CH_4_ emissions per unit of grassland area were significantly lower by 22.75%, 84.17%, 23.48%, 30.59%, and 22.78%, respectively (*P* < 0.05, Table 6). Lastly, in the LBT group, CH_4_ emissions, CH_4_/BW^0.75^, and CH_4_ emissions per unit of grassland area decreased significantly by 17.05%, 19.39%, and 17.05%, respectively (*P* < 0.05, Table 6).

**Table 6:** Effects of the addition of *Lycium barbarum* seed, residue and twigs on gas emissions from sheep.

Item	Treatments^1^				SEM	*P-*value
	CON	LBS	LBR	LBT		
CH_4_ emissions, g/d	15.38^a^	12.78^b^	12.53^b^	13.14^b^	0.262	<0.001
CH_4_/DMI, g/kg DMI/d	15.34^a^	8.49^b^	8.33^b^	14.06^ab^	0.779	<0.001
CH_4_/OMI, g/kg OMI/d	15.52	11.47	10.63	13.03	0.841	0.179
CH_4_/BW^0.75^, g/kg BW^0.75^ per d	1.07^a^	0.87^b^	0.87^b^	0.90^b^	0.026	0.005
CH_4_/DMD, g/kg BW^0.75^ per d	1.65^a^	1.19^b^	1.27^b^	1.66^a^	0.064	0.003
CH_4_/OMD, g/kg BW^0.75^ per d	1.51	1.66	1.34	1.38	0.064	0.306
CH_4_ emissions, g/d/m^2^	35.90^a^ × 10^−4^	29.81^b^ × 10^−4^	29.24^b^ × 10^−4^	30.67^b^ × 10^−4^	0.611	<0.001

CH_4_= methane; DMI = dry matter intake; OMI = organic matter intake; DMD = digestibility of dry matter; OMD = digestibility of organic matter; SEM = standard error of the mean.

Within the same row with different lowercase letters are significantly different (*P* < 0.05).

1 CON, the control group (no supplementation); LBS, 2.5% *L. barbarum* seeds group (22.5 g); LBR, 7.5% *L. barbarum* residue group (67.5 g); LBT, 2.5% *L. barbarum* twigs group (22.5 g); *n* = 6.

### 3.5 Equations to predict N excretion, energy and CH_4_ emissions

Nutrient intake and live weight gain were employed to predict CH_4_ emissions, CH_4_-E, FN emissions, UN emissions, FE, and UE emissions per unit of metabolic body weight in grazing sheep (Table 7). The factors DMI, NDFI, OMI, ADFI, EEI, CPI, GEI, and LWG could predict CH_4_-E, FE and UE emissions, and FN emissions per unit metabolic body weight of grazing sheep, among which the FE emission prediction equation had the best fitting degree (Eq. 3a-3g), and the removal of any factor had little effect on the fitting degree. The factors DMI, OMI, ADFI, CPI and LWG can be used to predict CH_4_ emissions per unit metabolic body weight of grazing sheep (Eq. 1a-1e), with DMI, OMI and LWG being the best fit (Eq. 1c). In addition, the best factors for predicting CH_4_-E emissions per unit metabolic body weight of grazing sheep were DMI, EEI, CPI, LWG, and NDFI (Eq. 2d), and the addition and removal of other factors had little effect on the degree of fit of the equation (Eq. 2a-2f). The factors that had the best fit for the prediction of UE emission per unit metabolic body weight of grazing sheep were DMI, CPI, EEI and GEI (Eq. 4e), and the equation predicted by EEI alone had the worst fit (Eq. 4h). The best fitting factors for the prediction equation of FN emission per unit metabolic body weight of grazing sheep were DMI, EEI, GEI, NDFI, and ADFI (Eq. 5c), and the addition and removal of any other factors had little effect on the Eq. fitting degree (5a-5f).

**Table 7:** Linear prediction of excreted N, energy excretion, and CH_4_ emissions from calves using nutrient intake parameters.

Parameters	Eq.	*R*^2^	*P-*value	Code
CH_4_ emissions, g/kg·BW^0.75^/d	*Y* = 0.659^1^_(0.281_^2^_)_ + 0.466_(0.654)_ LWG + 2.558_(0.011)_ DMI - 1.548_(0.059)_ OMI - 0.338_(0.043)_ CPI - 0.603_(0.176)_ ADFI	0.306	0.037	1a
	*Y* = 0.745_(0.269)_ + 0.448_(0.652)_ LWG + 1.810_(0.008)_ DMI - 1.450_(0.059)_ OMI - 0.222_(0.040)_ CPI	0.304	0.026	1b
	*Y* = 0.572_(0.169)_ + 0.476_(0.635)_ LWG + 1.549_(0.007)_ DMI - 1.352_(0.057)_ OMI	0.315	0.014	1c
	*Y* = 0.562_(0.181)_ + 0.451_(0.679)_ LWG + 0.245_(0.002)_ DMI	0.213	0.031	1d
	*Y* = 0.774_(0.085)_ + 0.470_(0.688)_ LWG	0.186	0.020	1e
CH_4_-E, MJ/kg·BW^0.75^/d	*Y* = 0.028_(0.009)_ + 6.914_(0.001)_ DMI - 5.306_(0.020)_ EEI + 0.866_(0.001)_ CPI - 0.217_(0.015)_ LWG + 0.381_(0.009)_ NDFI - 3.036_(0.077)_ GEI + 0.011_(0.001)_OMI + 0.272_(0.012)_ADFI	0.608	0.002	2a
	*Y* = 0.029_(0.008)_ + 7.000_(0.001)_ DMI - 5.114_(0.019)_ EEI + 0.870_(0.001)_ CPI - 0.219_(0.015)_ LWG + 0.712_(0.003)_ NDFI - 3.385_(0.070)_ GEI + 0.016_(0.001)_ OMI	0.632	<0.001	2b
	*Y* = 0.029_(0.008)_ + 7.035_(0.001)_ DMI - 5.088_(0.017)_ EEI + 0.868_(0.001)_ CPI - 0.219_(0.014)_ LWG + 0.708_(0.003)_ NDFI - 3.424_(0.063)_ GEI	0.653	<0.001	2c
	*Y* = 0.029_(0.008)_ + 5.073_(0.000)_ DMI - 6.662_(0.011)_ EEI + 0.843_(0.001)_ CPI - 0.196_(0.014)_ LWG + 0.839_(0.003)_ NDFI	0.654	<0.001	2d
	*Y* = 0.023_(0.007)_ + 6.425_(0.000)_ DMI - 7.122_(0.011)_ EEI + 0.733_(0.001)_ CPI - 0.175_(0.014)_ LWG	0.633	<0.001	2e
	*Y* = 0.019_(0.007)_ + 6.554_(0.000)_ DMI - 7.309_(0.011)_ EEI + 0.790_(0.001)_ CPI	0.618	<0.001	2f
	*Y* = 0.039_(0.006)_ + 6.169_(0.001)_ DMI - 6.335_(0.014)_ EEI	0.335	0.005	2g
FE excretion, MJ/kg·BW^0.75^/d	*Y* = 7.496_(0.494)_ GEI - 0.007_(0.059)_ - 4.770_(0.059)_ NDFI + 1.527_(0.074)_ ADFI-2.287_(0.130)_ EEI - 1.749_(0.006)_ DMI + 0.093_(0.006)_ CPI + 0.033_(0.098)_ LWG + 0.000_(0.009)_ OMI	0.886	<0.001	3a
	*Y* = 7.495_(0.456)_ GEI - 0.007_(0.057)_ - 4.770_(0.056)_ NDFI + 1.527_(0.072)_ ADFI - 2.287_(0.118)_ EEI - 1.748_(0.005)_ DMI + 0.093_(0.006)_ CPI + 0.033_(0.094)_ LWG	0.893	<0.001	3b
	*Y* = 0.003_(0.052)_ + 7.309_(0.437)_ GEI - 4.742_(0.055)_ NDFI + 1.511_(0.070)_ ADFI - 2.157_(0.113)_ EEI - 1.696_(0.005)_ DMI + 0.086_(0.006)_ CPI	0.898	<0.001	3c
	*Y* = 0.015_(0.049)_ + 7.575_(0.426)_ GEI - 4.863_(0.053)_ NDFI + 1.544_(0.069)_ ADFI - 2.243_(0.111)_ EEI - 1.726_(0.005)_ DMI	0.900	<0.001	3d
	*Y* = 5.611_(0.267)_ - 0.006_(0.046)_ GEI - 4.922_(0.054)_ NDFI + 1.419_(0.069)_ ADFI - 1.828_(0.108)_ EEI	0.897	<0.001	3e
	*Y* = 0.044_(0.035)_ + 3.667_(0.063)_ GEI - 4.451_(0.052)_ NDFI + 1.062_(0.067)_ ADFI	0.890	<0.001	3f
	*Y* = 0.069_(0.033)_ + 3.400_(0.056)_ GEI - 3.141_(0.017)_ NDFI	0.879	<0.001	3g
UE excretion, MJ/kg·BW^0.75^/d	*Y* = 0.020_(0.009)_ - 9.846_(0.019)_ EEI + 13.682_(0.072)_ GEI - 3.904_(0.001)_ DMI + 0.240_(0.001)_ CPI - 1.813_(0.009)_ NDFI + 1.140_(0.011)_ ADFI + 0.112_(0.014)_ LWG - 0.066_(0.001)_ OMI	0.613	0.002	4a
	*Y* = 0.020_(0.008)_ - 9.946_(0.017)_ EEI + 13.833_(0.066)_ GEI - 4.044_(0.001)_ DMI + 0.246_(0.001)_ CPI - 1.786_(0.008)_ NDFI + 1.134_(0.010)_ ADFI + 0.112_(0.014)_ LWG	0.637	<0.001	4b
	*Y* = 0.022_(0.008)_ - 9.502_(0.017)_ EEI + 13.194_(0.064)_ GEI - 3.864_(0.001)_ DMI + 0.223_(0.001)_ CPI - 1.689_(0.008)_ NDFI + 1.080_(0.010)_ ADFI	0.643	<0.001	4c
	*Y* = 0.025_(0.007)_ - 8.731_(0.015)_ EEI + 11.791_(0.059)_ GEI - 3.490_(0.001)_ DMI + 0.235_(0.001)_ CPI - 0.380_(0.003)_ NDFI	0.647	<0.001	4d
	*Y* = 0.027_(0.006)_ - 8.799_(0.015)_ EEI + 12.356_(0.056)_ GEI - 4.387_(0.001)_ DMI + 0.279_(0.001)_ CPI	0.658	<0.001	4e
	*Y* = 0.033_(0.005)_ - 9.081_(0.015)_ EEI + 13.632_(0.055)_ GEI - 5.173_(0.001)_ DMI	0.638	<0.001	4f
	*Y* = 0.032_(0.005)_ - 7.863_(0.017)_ EEI + 7.252_(0.038)_ GEI	0.554	<0.001	4g
	*Y* = 0.045_(0.004)_ - 0.624_(0.001)_ EEI	0.362	<0.001	4h
FN, g/kg·BW^0.75^/d	*Y* = 12.447_(6.972)_ GEI - 4.659_(0.813)_ NDFI - 0.238_(0.783)_ - 5.072_(0.085)_ DMI - 4.789_(1.833)_ EEI + 2.455_(1.044)_ ADFI - 0.084_(1.366)_ LWG - 0.285_(0.130)_ OMI	0.382	0.031	5a
	*Y* = 13.179_(6.425)_ GEI - 4.578_(0.789)_ NDFI - 0.252_(0.763)_ - 5.691_(0.077)_ DMI - 5.242_(1.681)_ EEI + 2.439_(1.018)_ ADFI - 0.084_(1.333)_ LWG	0.412	0.016	5b
	*Y* = 13.714_(6.147)_ GEI - 4.676_(0.769)_ NDFI - 0.379_(0.705)_ - 5.833_(0.075)_ DMI - 5.592_(1.600)_ EEI + 2.486_(0.995)_ ADFI	0.436	0.007	5c
	*Y* = 10.559_(5.875)_ GEI - 1.689_(0.288)_ NDFI + 0.118_(0.665)_ - 4.978_(0.077)_ DMI - 3.836_(1.545)_ EEI	0.386	0.009	5d
	*Y* = 5.650_(3.890)_ GEI - 1.772_(0.294)_ NDFI + 0.510_(0.619)_ - 3.816_(0.076)_ DMI	0.356	0.008	5e
	*Y* = 2.308_(0.789)_ GEI - 2.258_(0.242)_ NDFI + 0.055_(0.473)_	0.347	0.004	5f

*Y* = dependent variable; DMI = dry matter intake; OMI = organic matter intake; NDFI = neutral detergent fiber intake; ADFI = acid detergent fiber intake; FN = fecal nitrogen; UN = urinary nitrogen; GEI = gross energy intake; FE = fecal energy; UE = urinary energy; CH_4_-E = methane energy; LWG = the live weight gain; CPI = crude protein intake; EEI = ether extract intake.

1 Preceded by the parentheses is the normalization factor.

2 Inside the parentheses is the standard error.

## 4 Discussion

### 4.1 Supplementing feed with *L. barbarum* by products to grazing sheep: Effects on intake, digestion, and live weight gain of grazing sheep

In ruminant nutrition, the effectiveness of feed additives is closely linked to their bioactive composition and nutrient synergy (Patra et al., 2017). The varying responses among the LBS, LBR, and LBT groups stem from the distinct physicochemical properties and bioactive profiles of each byproduct (Table 1), which interact differently with rumen microbial ecosystems and host metabolism. Supplementation with *L.* *barbarum* has been shown to significantly enhance daily weight gain in beef cattle (31.5%) and improve CP (6.18%) and crude fat digestibility (30.77%) in lambs (Abdallah et al., 2019), positively affecting ruminant performance, immune function, and meat quality. In this study, the addition of LBS and LBR significantly increased the DMI of sheep, which is related to the active ingredients *L. barbarum* polysaccharides and flavonoids. Polysaccharides are rich in sweetness, improving the palatability of forage and inducing intake (Chen et al., 2020). Flavonoids (such as quercetin and other compounds) enhance the palatability of feed by masking bitter phytochemicals and activating olfactory receptors, thereby increasing voluntary DM and OM intake (Ahmed et al., 2021). Although LBT also contains the active ingredients polysaccharides and flavonoids, its DMI was even lower than that of the CON group, possibly due to its inherently higher fiber content affecting the texture and palatability of the feed, leading to reduced selective intake (Lourencon et al., 2024). Additionally, the high fiber content decreased the digestibility of roughage, slowed the passage rate of chyme, and produced a sense of satiety, further reducing nutrient intake. Similar to the changes in DMI, although no significant differences in OMI were observed among the groups, an improvement in OMI was still detectable in the LBS and LBR groups. Further analysis revealed that the intake and digestibility of NDF and ADF in sheep from the LBS and LBR groups were significantly increased. The intake depended on DMI, while the improvement in digestibility was attributed to various bioactive components. Studies have shown that supplementing diets of bulls (Araújo et al., 2015), lambs, and calves (Tu et al., 2015) with chitosan, mannan and β-glucan, and bee pollen polysaccharides, respectively, can enhance nutrient digestibility. Polysaccharides can stabilize rumen pH (Krause et al., 2003) and enhance cellulase activity, thereby improving fiber digestibility. Plant flavonoids have also been proven to enhance production performance, nutrient digestibility, and rumen fermentation (Ma et al., 2017). Flavonoids can enrich cellulolytic bacterial populations (e.g., *Ruminococcus flavefaciens*), improving nutrient bioavailability (Patra et al., 2017). In addition, betaine regulates lipid metabolism and growth factors, enhances feed conversion efficiency, stimulates metabolic activity, and promotes weight gain, particularly under thermal or osmotic stress (Eklund et al., 2005). Recent studies indicate that betaine can increase DMI and OMD in heat-stressed dairy cows by reducing energy expenditure on stress adaptation (Malik et al., 2024). It is noteworthy that the CPI in the LBS and LBR groups was higher than that in the CON and LBT groups. Additionally, nutrient intake per unit metabolic body weight (particularly CP) was higher in the LBR group than in the LBS group. This disparity is due to two factors: (1) the LBR group consumed a higher proportion of *L. barbarum* byproducts, which contained elevated levels of nutrients; and (2) the higher concentrations of bioactive compounds, specifically polysaccharides and flavonoids, in the LBR group likely played a significant role in regulating nutrient intake. Studies have shown that polysaccharides, as energy substrates for fiber- and protein-degrading bacteria, can improve N utilization efficiency and drive higher CPI (Abdallah et al., 2019; Qiang et al., 2023). Meanwhile, flavonoids with antioxidant properties can effectively prevent the oxidative degradation of CP and EE in the rumen, enhancing their utilization (Patra et al., 2017). However, the CP digestibility in the LBS group sheep unexpectedly decreased, which is similar to the results observed in dairy cows with dietary addition of rapeseed cake leading to reduced CP digestibility (Rinne et al., 2015). The speculated reasons may be that the CP in LBS was excessively protected by oil or that glucosinolates and their metabolites inhibited CP digestion. The specific reasons warrant further investigation (Gao et al., 2022). In addition, the EEI and digestibility in both the LBS and LBR groups were significantly higher than those in the CON and LBT groups. However, the LBR group exhibited lower nutrient digestibility (notably for EE) and LWG compared to the LBS group. These differences may be attributed to *L. barbarum* seed oil (LBSO), a lipid component present in the seeds. A limitation of this study was the lack of quantitative analysis of LBSO content in the supplemented byproducts, which could be a critical explanatory variable. Current evidence suggests that plant-derived lipids positively influence feed conversion efficiency and weight gain in ruminants by enhancing lipid metabolism and energy partitioning (Oliveira, 2020). LBSO can provide essential fatty acids, reduce heat stress, adjust rumination time, and promote digestive metabolism (Zhao et al., 2022). The differences in digestibility between the LBR and LBS groups may reflect an antagonistic interaction between the lipid components of the seed oil and polysaccharide-mediated digestive processes, warranting further targeted metabolomic and metagenomic investigations.

### 4.2 Supplementing feed with *L. barbarum* by products to grazing sheep: Effects on N metabolism

Nitrogen metabolism is of crucial significance for the growth and development of grazing sheep as well as environmental protection. Higher CP digestibility helps to reduce N loss and the risk of environmental pollution. Therefore, improving N use efficiency (NUE) has become an important topic in the field of ruminant nutrition research. In this study, compared to the CON group, the NI of sheep in the LBS and LBR groups significantly increased, which is closely related to the higher DMI. Additionally, the direct utilization of N-rich substances (such as CP) in the byproducts of wolfberry seeds and residues also led to an increase in NI (Wang et al., 2019a, Wang et al., 2019b). Conversely, the lower CP content in the byproducts of the LBT group and the lower DMI of sheep in this group resulted in a lower NI compared to the CON group. Additionally, the RN and DN of sheep in the LBS and LBR groups were significantly higher than those in the CON and LBT groups, which is attributed to the energy produced by the fermentation of carbohydrates easily degraded by rumen microorganisms in the byproducts, serving as the carbon source for microbial protein synthesis. Meanwhile, the N source (such as protein) in *L. barbarum* byproducts is degraded synchronously with carbohydrates, providing the necessary N source and carbon framework for microbial protein synthesis, thus improving the NUE (Lu et al., 2019; Bach et al., 2005). Additionally, various bioactive substances in the byproducts also play an important role, as studies have shown that plant polysaccharides can reduce the rumen ammonia N concentration, improve the rumen fermentation pattern and the ammonia N utilization rate, and improve NUE (Zhang et al., 2024). Dietary supplementation of sand onion polysaccharides reduces ruminal NH_3_–N concentration and improves N utilization in sheep, while supplementation of *Astragalus* polysaccharides improves ruminal fermentation pattern, improves volatile fatty acid (VFA) concentration, and increases ammonia N utilization in lambs (Zhong et al., 2012). In addition, some plant active ingredients, such as flavonoids, can inhibit protozoa. Protozoa will affect the hydrolysis and deamination of proteins, secrete deaminase to degrade feed proteins into NH_3_–N, but cannot use NH_3_–N to synthesize proteins and consume a large amount of bacterial proteins, reducing the amount of microbial proteins entering the duodenum (Oskoueian et al., 2013; Patra et al., 2012), thus improving the NUE. Research indicates that betaine alters intestinal morphology and microbial communities, improving the digestibility of nutrients by maintaining the homeostasis of intestinal cells. Consistent with our findings, betaine supplementation increased the production of microbial crude protein (MCP) in dairy cows (Shah et al., 2020). Additionally, supplementing lambs with 5 g/d of betaine enhanced feed efficiency, as well as DN and RN (Ren et al., 2022). However, studies have also shown that ammonia nitrogen in dairy cows decreases with the addition of betaine (Cheng et al., 2020; Wang et al., 2010). The differences may stem from dietary composition and the level of betaine supplementation. In addition, polysaccharide (yeast wall polysaccharide) was found to improve the gastrointestinal tract morphology of ruminants by increasing the length, width, and mucosal thickness of rumen epithelial cell papillae, increasing the crypt depth and the height of intestinal villi, thereby improving the nutrient absorption efficiency (Lesmeister et al., 2004), while the effect of *L. barbarum* polysaccharide on the gastrointestinal tract morphology of grazing sheep needs further research. Although no significant differences in FN, UN, FN/NI, and UN/NI were observed among the groups, the data indicate that FN in the LBS, LBR, and LBT groups increased compared to the CON group, while UN exhibited the opposite trend. Studies have shown that *L. barbarum* byproducts often contain a certain amount of dietary fiber. Dietary fiber can accelerate intestinal peristalsis and reduce the reabsorption time of N-containing substances in the intestine (Capuano, 2017; Yan et al., 2019). The N-containing substances that are not fully digested and absorbed will be excreted with feces, resulting in an increase in FN loss. Moreover, dietary fiber may entrap some N-containing compounds, making it difficult for them to dissociate and be absorbed in the intestine and finally excreted out of the body (Adams et al., 2018), which leads to higher FN excretion. The reduction in UN may be related to the active ingredient flavonoids, which can reduce the degradation of soluble proteins in the rumen by binding to proteins, thereby decreasing the concentration of ruminal ammonia nitrogen and reducing UN excretion (Ibrahim and Hassen, 2022). Previous studies have demonstrated that seabuckthorn flavonoids reduce UN excretion in fattening lambs and Altay sheep (Hao et al., 2023), which aligns with the current results. Further analysis found that the sheep in the LBR group had a higher N metabolism efficiency than those in the LBS group, as manifested by increased NI, DN, and RN. which can be attributed to two primary mechanisms: 1) a higher proportion of proteins or nitrogen-containing compounds in the LBR diet was directly utilized for nutritional purposes, and 2) the elevated content of bioactive components (e.g., polysaccharides and flavonoids) in the LBR regimen exerted multifaceted regulatory effects. These compounds enhanced nutrient intake (including DM consumption) and digestive efficiency through multiple metabolic pathways, while simultaneously promoting ruminal fermentation dynamics and optimizing microbial protein synthesis capacity. Furthermore, certain plant oils have been demonstrated to reduce ruminal ammonia-N concentration, decrease protozoal abundance, and mitigate UN loss in ruminants, thereby enhancing N utilization efficiency (Atikah et al., 2018; Fan et al., 2015; Liang et al., 2015). Considering the absence of significant differences in DN and RN between the LBS and LBR groups in the present study, it was hypothesized that LBSO does not exert a detrimental effect on N metabolism in sheep. Moreover, its impact appears to be less pronounced than that of bioactive compounds such as polysaccharides and flavonoids. Further in-depth investigation is warranted to elucidate the specific mechanisms of action.

### 4.3 Supplementing feed with *L. barbarum* by products to grazing sheep: Effects on energy metabolism

The energy in the diet is mainly used to maintain the normal life activities and production needs of animals, and determines the intake, digestion, and conversion efficiency of nutrients. In this study, compared with the CON group, the GEI of the sheep in the LBS and LBR groups significantly increased, which was related to the rapid fermentation and energy supply of the highly fermentable carbohydrates in *L. barbarum* seeds and residues. Microorganisms in the rumen degrade carbohydrates to produce volatile fatty acids and microbial proteins, meeting 70% to 80% of the body's energy requirements. The lower GEI, DE, and ME in the LBT group may be attributed to the combined effects of the high content of difficult-to-degrade cellulose and lignin in *L. barbarum* branches and leaves, along with the lower DMI intake in sheep. FE, UE, HP, and CH_4_-E are the main forms of energy loss in ruminants (Wei and Yang, 2019), while DE, ME, and NE reflect the efficiency and utilization of energy metabolism. Results indicated that DE, ME, and NE in the LBS and LBR groups were significantly higher than those in the CON group, with the active component polysaccharides and flavonoids playing an important role. Plant polysaccharides have been proven to regulate rumen function, stimulate microbial fermentation, increase the level of volatile fatty acids in the rumen, and improve energy metabolism and mitochondrial function by reducing the accumulation of free fatty acids and lactic acid in the blood (Eklund et al., 2005; Wang et al., 2019). Flavonoids can provide electrons for the three major cycles of glycolysis, the citric acid cycle, and oxidative phosphorylation, promoting redox reactions and facilitating energy metabolism pathways. Additionally, they can enhance the production of rumen VFA, thereby acting on CoA and promoting energy metabolism (Chen et al., 2015). Similarly, betaine has been demonstrated to enhance energy metabolism efficiency by improving feed efficiency and maintaining intestinal cell homeostasis. Studies have shown that dietary betaine supplementation reduces heat production and maintenance energy requirements in pigs, while increasing retained energy and CH_4_ production (Schrama et al., 2003). The addition of betaine to lamb diets has been found to improve the apparent digestibility of GE (Ren et al., 2022). The higher energy metabolic efficiency also explains the generally lower NE_m_ and ME_m_ results observed in the LBS and LBR groups of sheep in this study. It is noteworthy that the DE, ME, DE/GE, and ME/GE values of the LBS group were all higher than those of the LBR group, which may be related to the special component, LBSO, in the byproduct LBS. This component not only serves as a high-energy raw material for ruminants but also extends digestion time and promotes forage digestion, thereby enhancing the energy value of the forage (Nur Atikah et al., 2018). Studies have shown that the unsaturated fatty acids in LBSO are beneficial for the absorption of saturated fatty acids, promote body fat deposition, and reduce energy consumption (Nur Atikah et al., 2018). This also corroborates the findings of this study, where the LBS group exhibited lower FE, UE, FE/GE, and UE/GE compared to the LBR group. The prediction Eq. for FE and UE losses per unit of metabolic body weight of sheep showed that the GEI was the main factor affecting their emissions, and the linear relationship between GEI, FE, and UE also proves this. As the GEI increases, both gradually increase, which is also the reason for the increased FE loss in the supplemented groups. Supplementation with LBS, LBR, or LBT can significantly reduce the CH_4_-E of grazing sheep, which may be due to the inhibitory effect of the active ingredients in these byproducts on CH_4_ emissions (Zhang et al., 2022). These results are attributed to the changes in the microbial community structure caused by plant active ingredients and secondary metabolites, especially the inhibitory effect on methanogenic archaea.

### 4.4 Supplementing feed with *L. barbarum* by products to grazing sheep: Effects on greenhouse gas emissions

Ruminant fermentation produces greenhouse gases CO_2_ and CH_4_. Among them, the greenhouse effect potential of CH_4_ is as high as 28 times that of CO_2_, and its energy loss accounts for approximately 2% to 12% of the total energy intake. The increase in gas emissions leads to energy waste, reduced production efficiency, and ecological harm (Herrero et al., 2013). Therefore, reducing CH_4_ emissions is of great ecological and economic significance. Studies have shown that plant secondary metabolites and active compounds can reduce CH_4_ emissions (Min et al., 2020). Dietary supplementation of gallic acid and condensed tannins in Simmental cattle can significantly reduce CH_4_ emissions (Wei, 2017). In this study, the daily CH_4_ emissions and CH_4_ emissions per unit of metabolic body weight in the treatment groups were significantly reduced compared to the CON group, with the LBT group showing the smallest reduction (17.05%). This may be due to the higher lignin content in the byproducts potentially promoting CH_4_ emissions by reducing fermentation, which is similar to the findings of reduced CH_4_ emissions by supplementing grape pomace in dairy cow diets (Moate et al., 2014). The LBR group exhibited the largest reduction (22.75%), likely attributable to its higher supplementation level (7.5%) and synergistic bioactive compounds such as flavonoids directly inhibiting methanogen activity. Studies have shown that flavonoids can directly act on methanogens, interfere with their intracellular metabolic processes, inhibit their growth and the activity of enzymes related to CH_4_ production, and thus reduce CH_4_ production (Oskoueian et al., 2013). Similar to the results of this study, dietary supplementation of citrus flavonoids and garlic flavonoids has been proven to reduce CH_4_ emissions in dairy cows (Khurana et al., 2023), beef cattle (Bitsie et al., 2022), Holstein calves (Brand et al., 2021), and sheep (Ahmed et al., 2021). The most striking results appear in CH_4_/DMI and CH_4_/DMD, where the two indicators in the LBS and LBR groups of sheep are significantly lower than those in the CON and LBT groups. This serves as important evidence from a different perspective that the nutrient digestibility of sheep has improved, the enhancement in feed conversion rate leads to a reduction in CH_4_ production during the fermentation process, which is a manifestation of the improvement in rumen function by various active ingredients. Studies have shown that flavonoids and polysaccharides can change the composition and proportion of the rumen microbial community, inhibit protozoa such as rumen ciliates (Belanche et al., 2015; Cheong et al., 2023; Sun et al., 2022), shift the rumen fermentation pattern towards the production of propionic acid, competitively utilize hydrogen with methanogens, and inhibit the CH_4_ production pathway (Kim et al., 2013; Wei and Yang, 2019). Additionally, the CH_4_/DMD in the LBT group was unexpectedly higher than that in the CON group. This could be attributed, on one hand, to the lower addition level and active ingredient content, which had a weaker effect on rumen function and methanogens; on the other hand, the higher crude fiber and lignin content reduced digestibility and promoted CH_4_ emissions. Although the supplementation levels of byproducts and active ingredients in the LBS group were lower than those in the LBR group (similar to the LBT group), the LBS group still demonstrated a stronger CH_4_ reduction effect (especially CH_4_/DMD and CH_4_/BW^0.75^, which were even lower than those in the LBR group). This is related to the specific active ingredient, LBSO, present in the LBS group. Most studies have demonstrated that the incorporation of vegetable oils into the diet effectively reduces ruminal CH_4_ production, with similar effects observed irrespective of the fatty acid composition of the oils (Castagnino et al., 2014; Eugène et al., 2008; Jalč et al., 2006; Zhang et al., 2008). The presence of vegetable oils is linked to a decrease in methanogens and ciliate protozoa within the rumen (Broucek, 2018; Patra et al., 2017). Concurrently, the biohydrogenation of unsaturated fatty acids in oils competes with CH_4_ generation for hydrogen molecules, resulting in a substantial diversion of H_2_ away from methanogenesis towards fatty acid saturation (Toprak, 2015; Vargas et al., 2019). Consequently, the significant reduction in CH_4_ emissions observed in sheep from the LBS group may be attributed to the high concentration of LBSO. The most crucial point is that the results indicate the LBS, LBR, and LBT groups all demonstrated a significant potential to reduce CH_4_ emissions per unit of grassland area compared to the CON group, highlighting the dual benefits of *L. barbarum* byproducts: reducing emissions while maintaining grazing productivity, which is a key factor in sustainable livestock systems. The CH_4_ prediction equation indicates that DMI and OMI are the primary factors influencing CH_4_ emissions per unit of metabolic body weight in sheep. Specifically, the effect of DMI on CH_4_ emissions is positive, whereas the intake of other nutrients, such as OM and CP, has a negative impact on CH_4_ emissions. Higher nutrient content correlates with increased digestibility. Easily digestible forage alters the rumen fermentation process, shifting it towards the production of propionic acid (Olijhoek et al., 2018). High-digestibility forage also modifies the rumen microflora, enabling bacteria and fungi to utilize the forage more efficiently. As the population of these microorganisms increases, they inhibit the proliferation of archaea, including methanogens (Ma et al., 2025). Furthermore, high-digestibility forage enhances the efficiency of the rumen microbial respiratory chain, accelerates electron transfer, and reduces the accumulation of free hydrogen, but methanogens depend on hydrogen as a crucial electron donor for CH_4_ synthesis; thus, a reduction in hydrogen availability limits CH_4_ production (Greening et al., 2019; Olijhoek et al., 2018). In conclusion, all *L. barbarum* byproducts examined in this study demonstrated the potential to mitigate CH_4_ emissions, a phenomenon closely linked to their active ingredients.

## 5 Conclusion

*L. barbarum* byproducts exhibit distinct nutritional and regulatory properties. The differential impacts of various byproduct types on nutrient utilization and CH_4_ emissions in sheep arise from their unique bioactive compositions, physicochemical characteristics, and inherent interactions with rumen microbial-host systems. Supplementation with 2.5% *L. barbarum* seeds or 7.5% *L. barbarum* residue enhanced nutrient intake and digestibility, improved systemic energy and N utilization efficiency, promoted live weight gain, and reduced CH_4_ emissions. In contrast, the inclusion of 2.5% *L. barbarum* twigs showed negligible effects on live weight gain, while exhibiting substantial CH_4_ mitigation potential. These findings underscore the importance of by product-specific formulation strategies to synergistically optimize ruminant productivity and environmental sustainability.

## Credit Author Statement

**Xiaoyun Zhang:** Writing – review & editing, Writing – original draft, Software, Resources, Methodology, Formal analysis, Data curation. **Wuchen Du:** Writing – review & editing, Resources, Methodology. **Kaili Xie:** Resources, Methodology. **Lijuan Ran:** Resources, Methodology. **Wanhe Zhu:** Resources. **Fujiang Hou:** Writing – review & editing, Writing – original draft, Visualization, Validation, Supervision, Resources, Project administration, Methodology, Investigation, Funding acquisition, Conceptualization.

## Declaration of competing interest

We declare that we have no financial and personal relationships with other people or organizations that can inappropriately influence our work, and there is no professional or other personal interest of any nature or kind in any product, service and/or company that could be construed as influencing the content of this paper.
