# Supplementary material for: Supplementation with Lycium barbarum byproducts improves energy and nitrogen metabolism, and reduces methane emissions in sheep grazing alfalfa/tall fescue pastures
Source: Anim Nutr. 2025 Oct 6;23:206–19. doi: 10.1016/j.aninu.2025.08.003 (PMC12664073; doi:10.1016/j.aninu.2025.08.003)
Supplement: Multimedia component 1 [file mmc1.docx]

**Table S1**

Effects of test batch on dry matter intake (DMI), Feces and urinary excretion, and greenhouse gas emissions.

| Items | Batches | | | | SEM | *P-*value |
| --- | --- | --- | --- | --- | --- | --- |
|  | 1 | 2 | 3 | 4 |  |  |
| DMI, kg/d | 1.28 | 1.28 | 1.21 | 1.26 | 0.064 | 0.980 |
| Feces excretion, kg/d | 0.40 | 0.41 | 0.41 | 0.43 | 0.014 | 0.927 |
| Urine excretion, kg/d | 1.25 | 1.24 | 1.32 | 1.11 | 0.046 | 0.428 |
| Methane emission, g/d | 14.61 | 16.98 | 14.31 | 14.27 | 0.497 | 0.158 |
| Carbon dioxide emission, g/d | 823.65 | 839.81 | 776.31 | 811.30 | 19.478 | 0.724 |

SEM = standard error of the mean.
